# Prognostic value of normal regadenoson stress perfusion cardiovascular magnetic resonance

**DOI:** 10.1186/1532-429X-15-108

**Published:** 2013-12-21

**Authors:** Benjamin H Freed, Akhil Narang, Nicole M Bhave, Peter Czobor, Victor Mor-Avi, Emily R Zaran, Kristen M Turner, Kevin P Cavanaugh, Sonal Chandra, Sara M Tanaka, Michael H Davidson, Roberto M Lang, Amit R Patel

**Affiliations:** 1 Department of Medicine, Northwestern University, Chicago, IL, USA; 2 Department of Medicine, University of Chicago, Chicago, IL, USA; 3 Department of Medicine, University of Michigan, Ann Arbor, MI, USA; 4 Department of Radiology, University of Chicago, MC 5084, 5841 S Maryland Ave., Chicago, IL 60637, USA

**Keywords:** Cardiovascular magnetic resonance, Myocardial perfusion, Regadenoson

## Abstract

**Background:**

Regadenoson is a vasodilator stress agent that selectively activates the A_2A_ receptor. Compared to adenosine, regadenoson is easier to administer and results in fewer side effects. Although extensively studied in patients undergoing nuclear perfusion imaging (MPI), its use for perfusion cardiovascular magnetic resonance (CMR) is not well described. The aim of this study was to determine the prognostic value of a normal regadenoson perfusion CMR in patients with known or suspected coronary artery disease.

**Methods:**

Patients with known or suspected coronary artery disease were prospectively enrolled to receive perfusion CMR (Philips 1.5 T) with regadenoson. Three short-axis slices of the left ventricle (LV) were obtained during first pass of contrast using a hybrid GRE-EPI pulse sequence (0.075 mmol/kg Gadolinium-DTPA-BMA at 4 ml/sec). Imaging was performed 1 minute after injection of regadenoson (0.4 mg) and repeated 15 minutes after reversal of hyperemia with aminophylline (125 mg). Perfusion defects were documented if they persisted for ≥2 frames after peak enhancement of the LV cavity. CMR was considered abnormal if there was a resting wall motion abnormality, decreased LVEF (<40%), presence of LGE, or the presence of a perfusion defect during hyperemia. All patients were followed for a minimum of 1 year for major adverse cardiovascular event (MACE) defined as coronary revascularization, non-fatal myocardial infarction, and cardiovascular death.

**Results:**

149 patients were included in the final analysis. Perfusion defects were noted in 43/149 (29%) patients; 59/149 (40%) had any abnormality on CMR. During the mean follow-up period of 24 ± 9 months, 17/149 (11.4%) patients experienced MACE. The separation in the survival distributions for those with perfusion defects and those without perfusion defects was highly significant (log-rank p = 0.0001). When the absence of perfusion defects was added to the absence of other resting CMR abnormalities, the negative predictive value improved from 96% to 99%.

**Conclusion:**

Regadenoson perfusion CMR provides high confidence for excellent prognosis in patients with normal perfusion.

## Background

During the last twenty years, perfusion cardiovascular magnetic resonance (CMR) has evolved from an experimental diagnostic test performed in only a handful of academic centers to an established non-invasive technique for the detection of significant coronary artery disease. Compared to single-photon emission computed tomography (SPECT) perfusion imaging, perfusion CMR provides better spatial resolution without exposing the patient to ionizing radiation. In addition, the diagnostic accuracy of perfusion CMR is not limited by attenuation artifacts, which can confound the interpretation of perfusion SPECT, potentially leading to additional testing [1,2].

Currently, perfusion CMR is most commonly performed during hyperemia induced by adenosine [3]. A recent landmark trial comparing the diagnostic performance of perfusion SPECT with adenosine perfusion CMR reported superior sensitivity and negative predictive value of the latter [4]. Other studies have shown that one-year event-free survival in patients with a normal adenosine perfusion CMR is 98-100% [5-7].

While the diagnostic [8] and prognostic performance of adenosine perfusion CMR is excellent, adenosine requires weight-dependent dosing and is infused continuously via a separate intravenous line. In addition, adenosine is known to cause undesirable side effects, such as bronchospasm and AV nodal block. Regadenoson is a relatively new, potent vasodilator, but its pharmacokinetics allow for a simplified fixed-dose single bolus injection, and its selectivity of the cardiac A_2A_ adenosine receptor results in fewer side effects [9]. Indeed, regadenoson is quickly replacing adenosine as the preferred vasodilator for perfusion SPECT and has been used for clinical imaging in over 3 million patients [10].

Although there is a significant body of literature regarding the performance of regadenoson in perfusion SPECT [11-13], little is published about its use in patients undergoing perfusion CMR. Accordingly, in this study, we sought to 1) assess the safety and tolerability of regadenoson perfusion CMR, and 2) determine the prognostic value of a normal regadenoson perfusion CMR in patients with known or suspected coronary artery disease.

## Methods

### Study population and design

From July 2009 through September 2013, 176 patients referred for perfusion CMR for the evaluation of ischemia at a single academic center were prospectively enrolled. The University of Chicago Institutional Review Board approved the study, and each patient provided informed consent. Patients were excluded if they had an implantable cardioverter-defibrillator, pacemaker or other standard CMR contraindications, claustrophobia, severe reactive airway disease, high-grade AV nodal block, or GFR < 30 ml/min/1.73 m^2^. A 12-lead EKG was performed in all patients prior to CMR imaging to rule out high-degree AV nodal block. Patients were asked to stop all anti-anginal medications and caffeine-containing products 12 hours before the CMR exam. Each patient underwent a focused clinical evaluation in which baseline demographics, current symptoms, past medical history, and exercise capacity were assessed and medications documented.

After completion of the perfusion CMR study, patients were followed for a minimum of 1 year for the primary composite endpoint of coronary revascularization with percutaneous coronary intervention or coronary artery bypass grafting, non-fatal myocardial infarction (as documented by appropriate combination of symptoms, electrocardiography, and enzyme changes), and cardiovascular death (defined as fatal arrhythmias, myocardial infarction, or heart failure). Since the aim of this study was not to determine the diagnostic performance of regadenoson perfusion CMR, patients were referred for invasive coronary angiography based on the judgment of the patient’s referring physician. Follow-up was performed both by reviewing the medical records and by direct patient contact. Deaths were confirmed by the Social Security Death Index (SSDI). Primary event ascertainment was blinded to CMR findings.

### CMR Protocol

CMR images were acquired using a 1.5-T scanner (Achieva, Philips, Best, Netherlands) using a 5-element phased array cardiac coil. Retrospectively gated cine images were obtained using a steady-state free precession (SSFP) sequence (TR 2.9 ms, TE 1.5 ms, flip angle 60°, and temporal resolution ~40 ms). Standard long-axis views were obtained, including four-chamber, two-chamber, and three-chamber images. In addition, one series of short-axis slices (8 mm thickness, 2 mm gap) from base to apex was acquired.

In preparation for dynamic contrast-enhanced MPI imaging, three short-axis slices were selected representing the apex, mid, and basal left ventricular myocardium. Stress images were acquired approximately 60–90 seconds after the administration of regadenoson (Lexiscan, 0.4 mg I.V.; Astellas Pharma). During peak vasodilator stress, gadolinium-DTPA-BMA (Omniscan™, 0.075 mmol/kg at 4 ml/sec, I.V.) was given, and images were acquired for 50–70 consecutive heartbeats using a hybrid gradient echo/echo planar imaging sequence (voxel size ~2.5×2.5 mm, slice thickness 10 mm, flip angle 20°, repetition time 5.9 ms, echo time 2.5 ms, EPI factor 5, delay time 80 ms, and SENSE factor 1.3). Immediately after images were obtained, aminophylline 75–125 mg was injected intravenously. The same pulse sequence was repeated 15 minutes later to obtain perfusion images under resting conditions.

Late gadolinium enhancement (LGE) images of the same short- and long-axis views were obtained 5 minutes after the second injection of contrast using a T1-weighted gradient echo pulse sequence with a phase sensitive inversion recovery reconstruction (TR 4.5 ms, TE 2.2 ms, TI 250-300 ms, flip angle 30°, flip angle 5°, voxel size 2×2×10 mm, SENSE factor 2). An inversion time between 250 and 300 ms was used to achieve nulling of normal myocardium. Heart rate, blood pressure, and symptoms were monitored throughout the entire examination.

### CMR image analysis

Images were analyzed using commercial software (Philips ViewForum, Best, Netherlands). Short-axis slices were used to measure left ventricular end-diastolic (first cine phase of the R wave triggered acquisition) and end-systolic (image phase with the smallest ventricular cavity area in the majority of slices) volumes, mass, and ejection fraction by the Simpson method of disks [14]. All volumes and masses were indexed for body surface area. Detection of regional wall motion abnormalities was primarily based upon the short-axis view and confirmed in orthogonal long-axis views.

Perfusion images were visually assessed by a cardiologist with expertise in stress CMR and myocardial perfusion imaging (ARP). Stress-induced perfusion defects were defined as decreased signal intensity of the subendocardium in a coronary distribution during stress, involving ≥ 25% wall thickness, and persisting for ≥ 2 consecutive frames following peak enhancement of the left ventricular cavity. Resting images were used to exclude artifacts. LGE of the myocardium was also visually assessed and considered to be present if the signal intensity in the myocardium was greater than or equal to that seen in the blood pool in 2 adjacent slices or transverse imaging planes.

### Statistical analysis

Categorical variables were expressed as percentages and continuous variables as mean ± SD. Independent sample (unpaired) student’s *t-test* (equal variances not assumed) was used to compare the means of normally distributed continuous variables in those patients with and without adverse outcomes. The Mann–Whitney *U* test was used to compare the means for non-normally distributed variables. Categorical variables were compared using Fisher’s Exact test. P values <0.05 were considered statistically significant.

Survival analysis was accomplished using Kaplan-Meier curves, and differences between observed and predicted primary outcome distributions were assessed using the log-rank test. Three Kaplan-Meier graphs were generated. The first graph compared patients with perfusion defects to patients without perfusion defects. The second graph compared patients with resting CMR abnormalities (LGE, LVEF < 40%, and/or regional wall motion abnormalities) to patients with normal resting CMR exams. The third graph compared patients with and without a combination of perfusion defects and resting CMR abnormalities. All statistical analyses were performed using Stata/IC (version 12, StataCorp LP, College Station, Texas).

## Results

### Patient characteristics

A total of 176 patients were enrolled in the study. Five patients were unable to complete the CMR exam due to previously unknown claustrophobia and two due to technical problems during the examination. Four patients had non-diagnostic perfusion studies due to severe dark rim artifact or poor image quality due to shift in coil position and were excluded from the final analysis.

The remaining 165 patients successfully completed regadenoson perfusion CMR. All patients were followed using 1) telephone contact, 2) communication with their physician, and/or 3) medical record review. Sixteen patients were lost to follow-up during the mean follow-up period of 24 ± 9 months. Thirteen of these patients had a negative perfusion study and 3 patients had a perfusion defect. None of these patients were reported as deceased when cross-referenced against the SSDI.

Final analysis was performed on 149 patients. The majority of patients (97%) had one or more cardiac risk factors. The mean age was 56 ± 15 years, and the mean body mass index was 29 ± 7 kg/m^2^. Patients who met the primary composite endpoint were significantly older and were more likely to have hypertension and left ventricular dysfunction. Patient characteristics are listed in Table 1.

**Table 1:** Baseline patient characterictics

**Baseline**	**All patients (n = 150)**	**Adverse outcome (n = 17)**	**No adverse outcome (n = 133)**	**p-value**
Females	66 (44)	6 (35)	60 (45)	0.45
History of CAD	45 (30)	9 (53)	36 (27)	0.06
History of CHF	24 (16)	6 (35)	18 (14)	0.10
History of smoking	53 (35)	10 (59)	43 (32)	0.06
Diabetes mellitus	44 (29)	9 (53)	35 (226)	0.06
Hypertension	90 (60)	15 (88)	75 (56)	0.01
Hyperlipidemia	111 (74)	13 (76)	98 (74)	0.88
Stroke	10 (7)	1 (6)	9 (7)	0.93
Peripheral vascular disease	7 (5)	2 (12)	5 (4)	0.35
LVEF (%)	58.0 ± 11.7	50.1 ± 16.6	59.5 ± 10.5	0.04
BMI (kg/m^2^)	29.0 ± 7.0	25.2 ± 9.50	29.0 ± 6.3	0.13
Age (years)	56.0 ± 14.7	65.9 ± 10.8	54.4 ± 14.6	0.01

Eleven percent of the patients (17/149) reached the primary composite endpoint of MACE (Table 1). Of these patients, 15 received coronary revascularization and 2 had sudden cardiac death. Two patients who died of non-cardiac complications (1 of lymphoma and 1 of sepsis due to gangrene) were censored for MACE evaluation.

### Regadenoson perfusion CMR results

**Figure 1:**
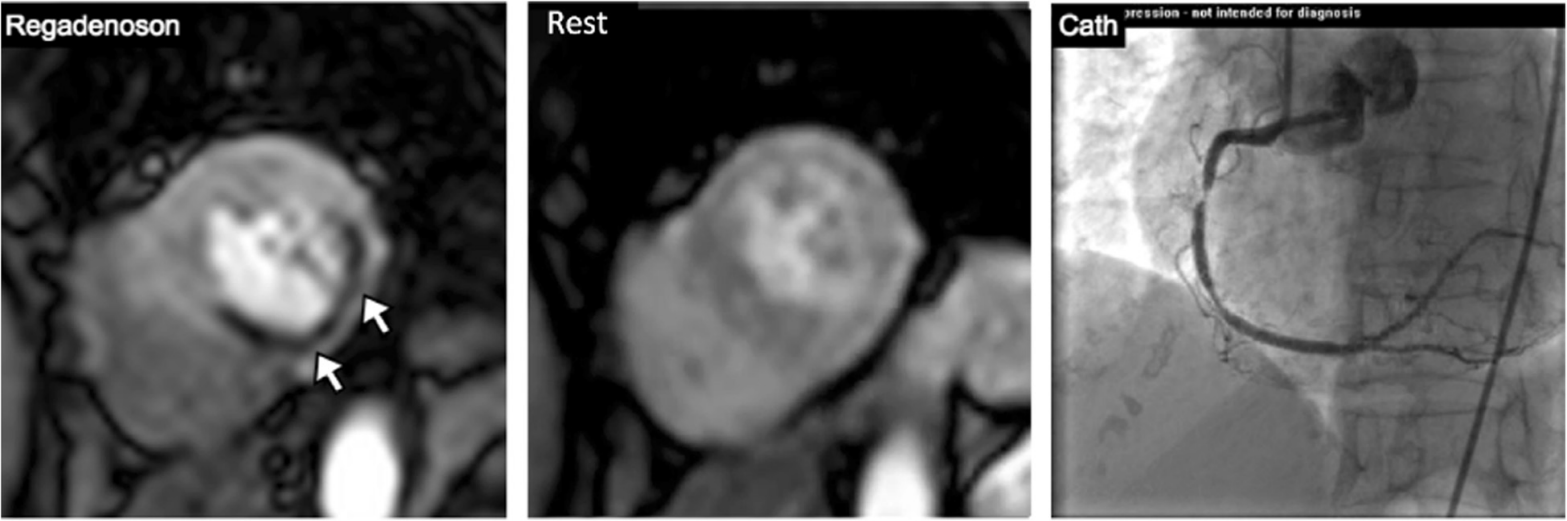
**Perfusion defect on regadenoson perfusion cardiovascular magnetic resonance.** Short axis image of a patient with extensive inferior, inferolateral, and inferoseptal wall hypoperfusion (white arrows) only during hyperemia. Subsequent cardiac catheterization in this patient reveals two significant stenoses in the proximal and mid-right coronary artery.

The average LVEF was 58 ± 12%. Eleven patients had an LVEF <40%. Perfusion defects during hyperemia were present in 43/149 (29%) of patients (Figure 1). Regional wall motion abnormalities were present in 20/149 (13%), and LGE was present in 33/149 (22%). Of the patients with evidence of LGE, 20 had LGE in a pattern consistent with prior myocardial infarction, and 13 had LGE in a pattern atypical for prior myocardial infarction. Atypical patterns of LGE consisted of focal areas of enhancement that did not involve the subendocardium or follow a coronary distribution.

### Hemodynamic effects of regadenoson

The hemodynamic effects of regadenoson administration are summarized in Table 2. Heart rate increased significantly by an average of 36 beats/min during hyperemia with regadenoson (p < 0.01). The maximal increase in heart rate occurred approximately 60 seconds after injection of regadenoson. A heart rate of >100 beats/min was noted in 60/149 (40%) patients, with a maximal heart rate of 151 beats/min. The mean systolic and diastolic blood pressure decreased slightly between baseline conditions and hyperemia. During rest perfusion, the average heart rate returned to near-baseline.

**Table 2:** Hemodynamic effects of regadenoson during CMR-MPI

**Hemodynamics**	**Baseline**	**Regadenoson**	**p-value (Baseline vs. Regadenoson)**	**Recovery**	**p-value (Regadenoson vs. Recovery)**
Heart rate (bpm)	69 ± 12	97 ± 15.0	<0.01	70 ± 12.4	<0.01
Systolic blood pressure (mmHg)	128 ± 19.4	126 ± 18.9	0.48	125 ± 18.4	0.48
Diastolic blood pressure (mmHg)	66 ± 12.3	62 ± 13.3	0.01	62 ± 12.1	0.96

### Safety and tolerability of regadenoson

**Figure 2:**
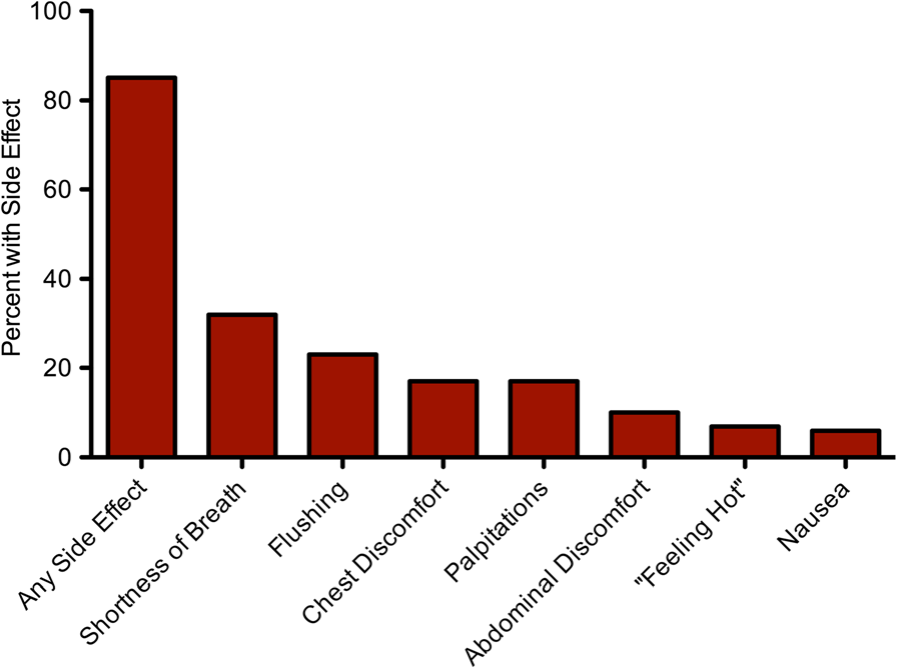
**Common side effects of regadenoson.** Percentage of patients who experienced one or more side effects after regadenoson administration.

There were no significant arrhythmias noted by rhythm strip monitoring during hyperemia. Two patients developed an infiltration of their intravenous line and returned on a later date to complete the exam. Overall, 127/149 (85%) of patients experienced symptoms associated with regadenoson administration (Figure 2). The four most common side effects included shortness of breath (32%), flushing (23%), chest discomfort (15%), and palpitations (15%). There was no evidence of bronchospasm. The majority of symptoms were reportedly mild and diminished by the end of the study. One patient experienced persistent headache and abdominal pain after the injection of regadenoson despite the administration of 125 mg of aminophylline. This patient had stress-induced perfusion defects and subsequently underwent coronary revascularization.

### Prognosis

**Figure 3:**
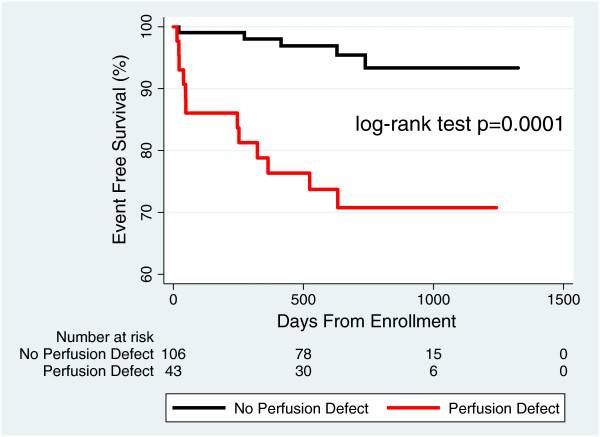
**Prognostic value of perfusion information.** Event-free survival for patients with perfusion defects compared to patients without perfusion defects.

**Figure 4:**
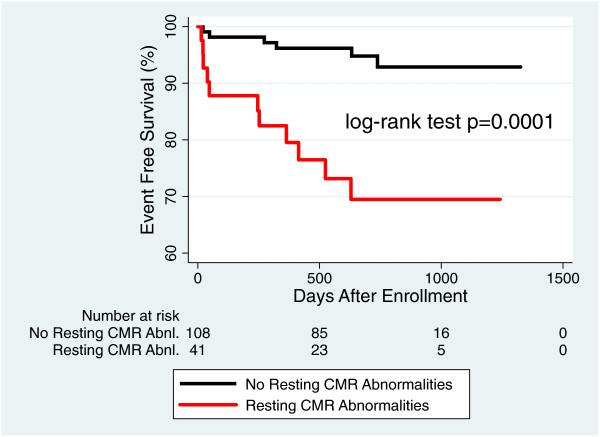
**Prognostic value of resting CMR abnormalities.** Event free survival for patients with resting CMR abnormalities (LVEF < 40%, presence of LGE, and regional wall motion abnormalities) compared to patients without CMR abnormalities.

**Figure 5:**
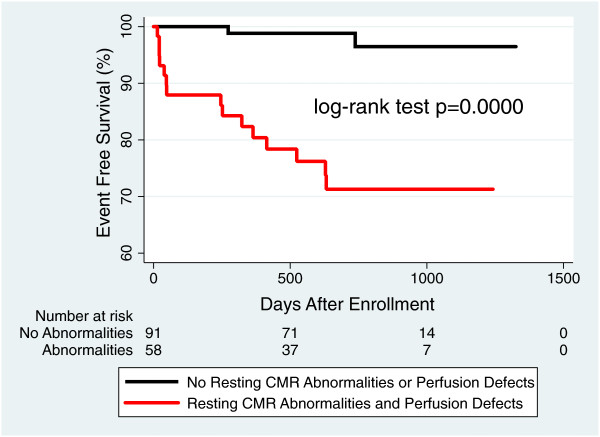
**Incremental prognostic value of adding perfusion data to other CMR findings.** Patients with resting CMR abnormalities and stress perfusion defects compared to patients without CMR abnormalities or perfusion defects. CMR = cardiovascular magnetic resonance; LVEF = left ventricular ejection fraction; LGE = late gadolinium enhancement.

During a follow-up of 24 ± 9 months, the primary composite endpoint occurred in 5/106 (4.7%; 2.4%/year) patients with no evidence of perfusion defect on regadenoson perfusion CMR study. The absence of perfusion defects on regadenoson perfusion CMR resulted in a negative predictive value of 96%, and the separation in the survival distributions for those with perfusion defects and those without perfusion defects was highly significant (Figure 3). When the absence of perfusion defects was added to the absence of other resting CMR abnormalities (LVEF < 40%, regional wall motion abnormalities, and presence of LGE), the negative predictive value improved from 96% to 99%. Kaplan Meier analysis shows an increasingly significant separation in survival curves between patients with and without CMR resting abnormalities alone (Figure 4) and patients with and without CMR resting abnormalities plus perfusion defects (Figure 5).

The presence of perfusion defects on regadenoson perfusion CMR was a statistically significant predictor of the primary composite endpoint (hazard ratio 6.4; 95% CI 2.25-18.2; p = 0.001). LVEF < 40% (hazard ratio 6.4; 95% CI 2.28-18.5, p = 0.001), the presence of LGE (hazard ratio 3.45; 95% CI 1.33-8.95, p = 0.011), and the presence of any resting regional wall motion abnormality (hazard ratio 3.12; 95% CI 1.10-8.88, p = 0.033) were also statistically significant for predicting the primary composite endpoint.

## Discussion

We studied the safety and tolerability of regadenoson perfusion CMR and determined the prognostic value of a normal study. Our results revealed that regadenoson perfusion CMR can be performed safely and its side effects were tolerated by nearly all (99%) of our patients. This study demonstrated that, not only does a normal regadenoson perfusion CMR identify a cohort of patients who are unlikely to have MACE during the subsequent 24 ± 9 months, but a strategy that incorporates assessment of regadenoson-induced perfusion defects with resting CMR abnormalities (LVEF < 40%, presence of LGE, regional wall motion abnormalities) provides a negative predictive value of 99%.

### Regadenoson characteristics

Regadenoson is the first selective vasodilator approved by the FDA and has comparable vasodilator efficacy when compared to adenosine [15]. Unlike adenosine, studies have shown that regadenoson, because of its highly selective A_2A_ receptor-binding properties, results in milder side effects, fewer cases of bronchospasm, and rarely any instances of high-degree AV nodal block [10-12,16].

In the ADVANCE MPI Phase III trials evaluating the effects of intravenous regadenoson in humans undergoing perfusion SPECT, the investigators compared a summed symptom score of flushing, chest pain, and dyspnea in patients receiving both adenosine and regadenoson and found that regadenoson resulted in significantly lower overall scores [12,16]. In addition, the same investigators developed tolerability questionnaires asking how comfortable patients felt with each drug and found that patients were significantly more comfortable with regadenoson compared to adenosine. Furthermore, no major adverse events occurred with the use of regadenoson in any of these studies.

Consistent with these studies, we found that the majority of patients experienced only mild side effects including shortness of breath, chest pain, palpitations, and flushing, which subsided by the end of the CMR exam. Although studies have reported a greater incidence of gastrointestinal symptoms and headaches with regadenoson compared to adenosine, we found these symptoms to be rare. In addition, there were no episodes of bronchospasm, life-threatening arrhythmias, or any other MACE during or within 24 hours of the test.

Furthermore, the simplicity of regadenoson administration makes it an attractive vasodilator to use in the CMR environment. Due to its longer half-life (33–108 min vs. 2–10 sec) [9] and the fact that neither its volume of distribution nor its clearance is significantly affected by body weight [17], regadenoson can be injected as a fixed-dose unit bolus. This decreases the chances of weight-dependent dosing errors and obviates the need for a second intravenous line or a CMR-compatible infusion pump.

### Prognostic value of normal regadenoson perfusion CMR

In our study, only 5/106 (4.6%; 2.4%/year) patients with normal perfusion CMR subsequently underwent coronary catheterization. None experienced myocardial infarction or cardiac death. Two patients, who initially presented with atypical chest pain, returned less than 1 year after a normal perfusion CMR test with typical angina. Two patients had multivessel coronary artery disease and 1 patient had end-stage liver disease and a significant coronary lesion.

It has been previously shown that the burden of ischemia may be significantly underestimated by visual assessment of perfusion defects in patients with multi-vessel coronary artery disease [18]. In addition, the diagnostic performance of vasodilator myocardial perfusion imaging in patients with liver disease is questionable given the underlying hyperemic state of these patients [19]. Regardless, the negative predictive value of 96% is consistent with event-free survival rates in patients with normal adenosine perfusion CMR [5-7,20-22] and the separation in the survival distributions for those with perfusion defects and those without (Figure 3) was highly significant (log-rank p = 0.0001).

### Added prognostic value of regadenoson MPI to a comprehensive resting CMR study

One of the most powerful attributes of CMR imaging in patients with known or suspected coronary artery disease is that it provides information on a multitude of known prognostic parameters including myocardial scar/viability, LV systolic function, and wall motion abnormalities. These parameters clearly help in identifying patients who are at risk for MACE. This study found that the addition of regadenoson perfusion imaging to these other parameters improves the prognostic power of CMR increasing the negative predictive value from 96% to 99%. Kaplan-Meier analysis shows that the combination of perfusion data to CMR resting assessment improves identification of those patients who will have MACE in the next 24 ± 9 months and those who will not (Figure 5). The incremental value of regadenoson perfusion CMR is that a greater number of patients without perfusion defects can be reassured about their cardiovascular future.

It has previously been shown that the addition of adenosine perfusion CMR to a comprehensive CMR study significantly enhances risk stratification. Steel et al. reported that the absence of reversible perfusion defects combined with the absence of LGE provided the lowest annual event rate compared to either finding in isolation [20]. A recent study examined the incremental prognostic value of cardiac clinical risk factors, LGE, LVEF, and aortic blood flow to adenosine perfusion in over 900 patients and found that each variable provided valuable complimentary information [23]. The absence of LGE and perfusion defects along with a normal LVEF ensured the lowest cardiac event rate. Our results are in line with these previous publications and extend their findings to a wider range of vasodilators.

### Technical considerations when using regadenoson perfusion CMR

While regadenoson did not significantly affect blood pressure, the average heart rate increased by 36 bpm, and 40% of patients developed tachycardia. Since the blood pressure decreased only minimally, the mechanism of regadenoson-induced tachycardia is thought to be secondary to the direct release of norepinephrine and epinephrine through stimulation of post-ganglionic receptors, rather than a strictly baroreflexive response [24]. Other studies have also noted a 21–27 bpm increase in heart rate with regadenoson [10-12,15,16,25], and several of these studies reported that the change in heart rate was significantly higher than that seen with adenosine [12,15,16]. Although the change in heart rate is typically well-tolerated by patients, fast heart rates can exacerbate dark rim artifacts and may prevent image acquisition during every heart beat making image interpretation more challenging [26]. This might be a particularly challenging issue in patients with elevated resting heart rates.

Another technical issue concerns the longer half-life of regadenoson and the stress-rest protocol we performed. Studies have shown that 0.4 mg of regadenoson will continue to induce a greater than 2-fold increase in coronary blood flow for up to 8.5 minutes [25]. When 100 mg of aminophylline was given, the hyperemia was dramatically blunted without any effect on the heart rate. However, as we have shown in a previous study, even after giving each patient 125 mg of aminophylline after stress perfusion imaging, some vasodilator effects of regadenoson remain during acquisition of the resting images [27].

Finally, although in perfusion SPECT, radioactive tracer is usually injected approximately 20 seconds after regadenoson is given, maximal coronary blood flow was reported to occur, on average, 102 seconds after regadenoson administration [28]. We, therefore, started perfusion imaging approximately 60–90 seconds after regadenoson was injected. This allowed enough time to administer the medication and return to the control room to initiate scanning.

### Limitations

This study has several limitations. First, coronary revascularization drove the majority of clinical endpoints and the decision to proceed with catheterization was likely influenced by the results of the perfusion CMR test. However, while the presence of perfusion defects may have influenced the decision to refer the patient for coronary angiography, the decision to revascularize was made at the time of the catheterization. Furthermore, we have shown that a significant number of patients without perfusion defects did well and only rarely revascularized over an average of 2 years and, thus, avoided unnecessary invasive testing. Second, we did not determine the sensitivity or specificity of regadenoson perfusion CMR to detect coronary stenoses, since only a small group of patients underwent coronary angiography. Third, although every patient was followed for a minimum of 1 year and had known or suspected coronary artery disease, there were a low number of cardiovascular events in this study. Finally, although aminophylline was given only to reverse the hyperemic effects of regadenoson, it is likely that it also helped mitigate some of the side effects. However, even before we administered the aminophylline, many patients reported that their symptoms had already started to abate.

## Conclusion

Vasodilator perfusion CMR is increasingly being used to evaluate patients with known or suspected coronary artery disease. Currently, adenosine is the most commonly used vasodilator, but it is cumbersome to administer and can be difficult for some patients to tolerate. We have demonstrated that, not only is regadenoson safe, well-tolerated, and easy to administer, but the prognostic performance of a negative regadenoson perfusion CMR study is comparable to that previously described for adenosine perfusion CMR. Furthermore, the addition of regadenoson stress imaging to information regarding LVEF, LGE, and regional wall motion further improves identification of those patients at lowest risk for future cardiac events. Given these characteristics, regadenoson is a reasonable and attractive alternative to adenosine for vasodilator stress perfusion CMR studies.

## Abbreviations

CMR: Cardiovascular magnetic resonance; SPECT: Single photon emission computed tomography; MPI: Myocardial perfusion imaging; MACE: Major adverse cardiovascular events; GFR: Glomerular filtration rate; SSFP: Steady state free precession; LGE: Late gadolinium enhancement; LVEF: Left ventricular ejection fraction; CI: Confidence interval.

## Competing interests

This study was supported by a grant from Astellas Pharma, Deerfield, Illinois.

## Authors’ contributions

BHF and NMB were responsible for acquiring, analyzing, and interpreting the data. BHF drafted the initial manuscript and NMB helped edit the final paper. VMA helped with statistical analysis and edit the final manuscript. ERZ, KMT, PC, AN, KPC, SC, SMT, and MHD all helped supply, recruit, and consent patients. In addition, they helped acquire all hemodynamic and MRI data. All were responsible for editing the manuscript. RML and ARP helped with concept and design of study as well as interpretation of data. Both RML and ARP helped edit the manuscript. All authors have read and given final approval of the version to be published.
